# Gynecological Cancers Caused by Deficient Mismatch Repair and Microsatellite Instability

**DOI:** 10.3390/cancers12113319

**Published:** 2020-11-10

**Authors:** Madhura Deshpande, Phillip A. Romanski, Zev Rosenwaks, Jeannine Gerhardt

**Affiliations:** 1 The Ronald O. Perelman and Claudia Cohen Center for Reproductive Medicine, Weill Cornell Medicine, New York, NY 10021, USA; mgd2002@med.cornell.edu (M.D.); par9114@med.cornell.edu (P.A.R.); zrosenw@med.cornell.edu (Z.R.); 2 Department of Obstetrics and Gynecology, Weill Cornell Medicine, New York, NY 10021, USA

**Keywords:** microsatellite instability, deficient mismatch repair, gynecological cancers

## Abstract

**Simple Summary:**

Microsatellite instability (MSI) has been detected in multiple types of gynecologic cancers. MSI is linked to mutations in mismatch repair (MMR) genes that cause mismatch repair deficit (dMMR) in human cells. Discovery of new therapeutic approaches are needed especially for treatment of advanced endometrial and other gynecological cancers with dMMR/MSI. In addition, there is a need to identify markers for reliable detection of dMMR/MSI gynecological cancers. Determination of the mechanism leading to these malignancies would help in diagnosis and therapeutic intervention. In this review, we summarize the MMR defects and MSI observed in gynecological cancers, and new therapeutic strategies to treat these cancers.

**Abstract:**

Mutations in mismatch repair genes leading to mismatch repair (MMR) deficiency (dMMR) and microsatellite instability (MSI) have been implicated in multiple types of gynecologic malignancies. Endometrial carcinoma represents the largest group, with approximately 30% of these cancers caused by dMMR/MSI. Thus, testing for dMMR is now routine for endometrial cancer. Somatic mutations leading to dMMR account for approximately 90% of these cancers. However, in 5–10% of cases, MMR protein deficiency is due to a germline mutation in the mismatch repair genes *MLH1*, *MSH2*, *MSH6*, *PMS2*, or *EPCAM*. These germline mutations, known as Lynch syndrome, are associated with an increased risk of both endometrial and ovarian cancer, in addition to colorectal, gastric, urinary tract, and brain malignancies. So far, gynecological cancers with dMMR/MSI are not well characterized and markers for detection of MSI in gynecological cancers are not well defined. In addition, currently advanced endometrial cancers have a poor prognosis and are treated without regard to MSI status. Elucidation of the mechanism causing dMMR/MSI gynecological cancers would aid in diagnosis and therapeutic intervention. Recently, a new immunotherapy was approved for the treatment of solid tumors with MSI that have recurred or progressed after failing traditional treatment strategies. In this review, we summarize the MMR defects and MSI observed in gynecological cancers, their prognostic value, and advances in therapeutic strategies to treat these cancers.

## 1. Introduction

Mismatch repair (MMR) deficiency leading to microsatellite instability (MSI) and malignancy have been identified in over 20 cancer types, including gynecological cancers [1]. Bonneville and colleagues found that over 30% of patients with endometrial cancer have high MSI. Other gynecologic malignancies in which MSI has been identified include uterine carcinosarcoma (3.5%), cervical squamous cell carcinoma and endocervical adenocarcinoma (2.6%), and ovarian high-grade serous carcinoma (1.4%) [1]. Endometrial cancer is the most common cancer type amongst the gynecological malignancies and the fifth leading cause of death among cancer patients in the world. Early stage endometrial cancer has an excellent prognosis. However, advanced and recurrent endometrial cancer have a poor survival prognosis making it imperative to better understand the disease etiology of endometrial cancer and other gynecological cancer types.

Microsatellites are DNA sequences composed of short nucleotide segments (~1–10 nucleotides, also known as short tandem repeats (STRs)) which repeat sequentially. Due to their repetitive nature, these DNA segments are prone to DNA polymerase pausing and slippage during DNA replication, which can result in mutations [2]. Microsatellites are susceptible to mutations, with a high range of 10^−6^ to 10^−2^ per generation [3]. DNA repair systems, such as MMR, exists to proofread the newly replicated DNA and to repair DNA mutations [4]. When these mutations occur within microsatellite regions causing deletion or expansions of the repetitive DNA sequences, this is referred to as MSI.

MSI is the consequence of an impaired MMR due to mutations in the MMR gene. MMR deficiencies can occur through either germline or somatic mutations. A germline mutation of one of the inherited MMR genes (*MLH1*, *MSH2*, *MSH6*, or *PMS2*) or deletion of the stop codon of the *EPCAM* gene causes the autosomal dominant condition, Lynch syndrome (also called hereditary nonpolyposis colorectal cancer) [5]. In addition, epigenetic alterations, such as DNA methylation in the gene promotor region can suppress transcription and interfere with the expression of MMR genes [6,7]. This includes hypermethylation of the *MLH1* promoter, epigenetic inactivation of *MSH2* [8], or downregulation of MMR genes by microRNAs [9]. Sporadic MMR deficiency (dMMR)/MSI is most commonly due to hypermethylation of the *MLH1* promoter region [10].

Cancer development due to dMMR/MSI is triggered by mutations in genes that contain microsatellites and are important for tumor suppression, such as *TGFbeta RII*, *IGFIIR*, *BAX*, *hMSH6*, and *hMSH3* genes. These genes contain short tracts of mononucleotide repeats in their coding sequences which makes them prone to mutations. It has been demonstrated that MMR deficient gynecological cancer cells also accumulate mutations in repeat sequences of cell growth, pro-apoptotic, cell regulatory, DNA repair, and oncogenes.

## 2. Why Microsatellites Are Hot-Spots for Genomic Instability?

Microsatellites are highly polymorphic and have high mutation rates (up to 10^−3^ mutations per locus per generation) [11]. Several mechanisms have been suggested as causes for the high mutation rate of microsatellites, including errors during DNA recombination, DNA repair, as well as DNA polymerase slippage during DNA replication [12,13,14,15]. However, recombination events have been shown to be a minor source of microsatellite variability [16,17]. It was reported that most short insertion mutations derive from a slippage-like process during DNA replication [18]. This indicates that these repeats are very much vulnerable to replication stress and error-prone replication which would explain the high mutation rate [19].

Several models propose how mispairing and slippage occur at microsatellites during DNA replication. It is known that repetitive DNA sequences like microsatellites are a challenge to the replication machinery, because these DNA sequences, when single-stranded, are able to form secondary non-B DNA structures like hairpins, triplexes, and cruciform structures, that can hinder DNA replication fork progression [11,20,21,22,23,24,25]. The consequences of replication fork stalling and interrupted polymerase activity depend upon the location where the secondary structures are formed. For example, it was found that the stability of tandem repeats depended on their orientation relative to the nearest replication initiation site [20]. Secondary DNA structures are more likely to form during lagging strand synthesis in the single strand Okazaki initiation zone. Indeed, several plasmid-based and ectopic studies in bacteria, yeast, and mammalian cells using repeat-containing constructs show that formation of secondary structures and subsequent DNA polymerase slippage during lagging-strand synthesis leads to repeat expansion [21,22,23,24,25,26,27]. If DNA polymerase stalling causes synthesis of additional repeats in the nascent strand, then misalignment results in an increase in repeat length and expansions. While formation of secondary repeat structures on the template strand can cause the polymerase to skip these repeats leading to repeat contractions [11,28,29,30]. 

Microsatellite repeats are able to form several different secondary non-canonical DNA structures. For example, poly(A)/poly(T) mononucleotide repeats are reported to form hairpin structures and are also able to undergo a stoichiometric transition into triple-stranded DNA structures (DNA triplexes, H-DNA) [31]. A genome-wide analysis of microsatellites has shown that poly(A) or poly(T) repeats are more abundant than poly(C) or poly(G) in all chromosomes [32]. Dinucleotide repeats that are inverted (e.g., (AT)n and (CG)n) are able to form cruciform and hairpin structures [33]. Trinucleotide repeats are observed to adopt hairpin, triplex, or quadruplex structures, depending upon the type of DNA sequence [34]. In addition, repeat sequences containing at least four stretches of two or more adjacent guanine nucleotides are able to form structures termed G-quadruplexes [15]. Dinucleotide repeats are the second most common repeats, though there is no bias in occurrence of any particular repeat. Trinucleotide alleles are approximately three-fold less abundant than di- and tetranucleotide repeats [35].

All these repeats can form secondary DNA structures that can pause DNA polymerase and hinder DNA synthesis. DNA replication was observed to stall within mono-, di-, tri-, and tetranucleotide microsatellites and the severity was dependent upon the sequence composition of the microsatellites [35]. Besides the DNA sequence, the type of DNA polymerase seems to play a very important role in replication fork stalling. For instance, Hile and Eckert reported that DNA polymerase kappa was stalled due to triplex DNA formation, which led to interrupted mutations within mononucleotide microsatellites [36]. Similarly, GA or GAA repeats (capable of adopting triplexes such as H-DNA) can cause stalling of DNA replication [37,38,39]. The degree of the sequence-specific replication fork stalling and the impact on the correct replication of various common microsatellites has to be determined. For example, Baptiste and colleagues compared the effects of several mammalian DNA polymerases on mononucleotide mutagenesis. They reported that there was a bias towards mononucleotide deletions [40].

Some tumor suppressors, pro-apoptotic genes, and oncogenes contain microsatellites and MSI at these genomic regions is associated with several types of cancers (Table 1 and Table 2). For example, mutations in short palindromic sequences were observed in the *p53* gene in 21% of patients with ovarian cancer [41]. DNA deletions and insertions in the *p53* genes were attributable to each of the following mechanisms: Mononucleotide runs, repeats of short tandem sequences, palindromes (inverted repeats of dyad symmetry), and runs of four or more purines or pyrimidines. Almost all deletions and insertions can be explained by one or more of these DNA sequences. The most common DNA sequence motifs seen at the site of deletions or insertions were runs of two to five consecutive mononucleotides.

It was also reported that in the *c-myc* gene, the P1 promoter and 3’ downstream breakpoint region contains multiple mixed GT and GC repeats [42] that are potentially able to form a Z-DNA. Z-DNA-forming CG repeats can cause small deletions within the repeats, likely due to slippage events during replication and can induce DSBs within or surrounding the repeats in mammalian cells, resulting in large-scale deletions. Alterations in the DNA sequence at the *c-myc* gene are reported to be associated with the development of uterine cervical cancer [43]. Similarly, Toyama and colleagues’ studies have put forth the relationship between MSI and *c-myc* amplification in human breast cancers [44]. In addition, the presence of CAG repeats in the *androgen receptor* (*AR*) gene was observed to increase the risk of ovarian cancer in the African American group [45]. Another example is the pro-apoptotic *Bax* gene that contains a tract of eight consecutive (G)8, and frameshift mutations in the (G)8 mononucleotide repeat are common in endometrial carcinomas with MSI [46]. It was suggested that BAX frameshift mutations play a key role in the process of tumor progression. *TGFbeta RII* is reported to harbor a poly(A) tract at codons 125–128 of its open reading frame, which is prone to slippage-related frameshift mutations [47]. Gain or loss of the *TGFbeta* pathway and its components are known to lead to a variety of diseases, including cancer.

Similarly, a study on endometrial cancer with MSI reported a higher frequency of mutations in the *PTEN* gene [46,48]. It has been shown that PTEN gene inactivation, mainly due to mutations, plays a pivotal role in tumor progression. This inactivation occurred in 24% of cases through frameshift mutations in polyA/T repeats. The frameshift and nonsense mutations, cause expression of proteins that have been reported to have null activity or less protein stability. In addition, Bilbao et al. studied mononucleotide microsatellites in genes involved in DSB repair and their role in endometrial cancer with MSI. They found mutations in several DSB repair genes, such as *RAD50, MRE11, ATR, BRCA1, CtIP,* and *MCPH1* suggesting that mutations in multiple genes of the DSB repair pathway are mutated in endometrial cancer with MSI [49].

## 3. The MMR Repair Pathway and Its Kryptonite

**Figure 1:**
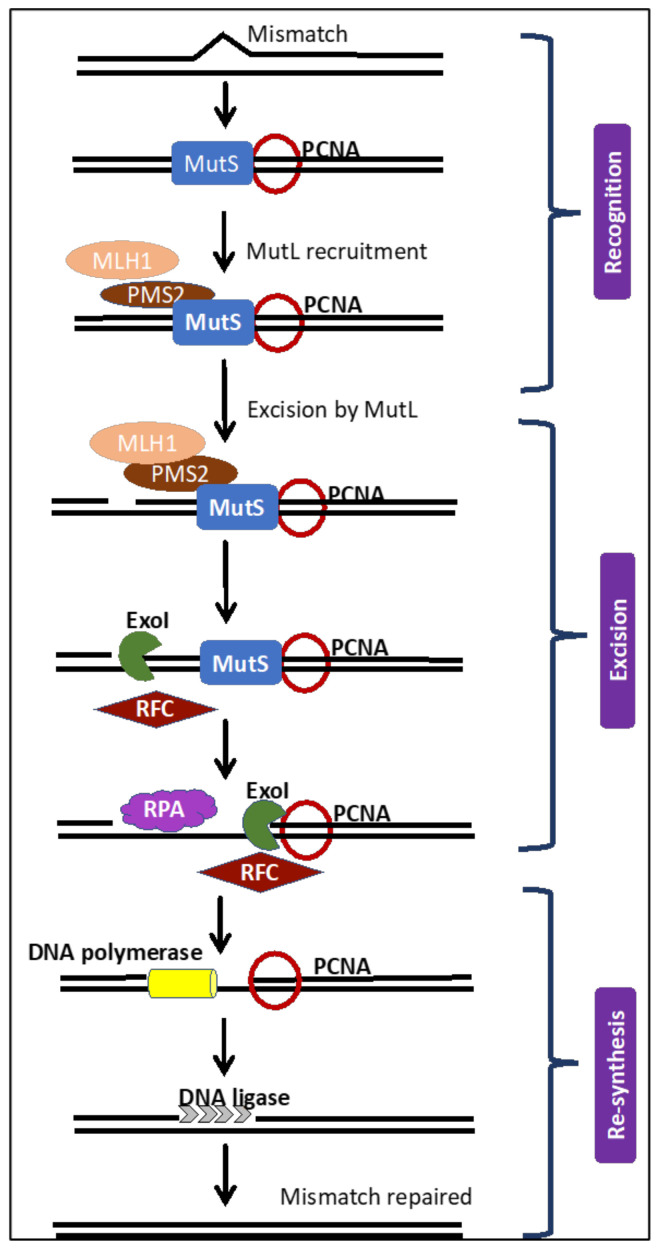
The mismatch repair (MMR) pathway that functions to correct errors in microsatellites. Schematic of the MMR pathway describing the three vital steps. (i) recognition of the mismatch by MutS complex, followed by recruitment of proliferating cell nuclear antigen and replication factor C, (ii) excision of the mismatched base(s) by MutL, and, finally, (iii) re-synthesis of the strand. The MMR system functions to correct errors introduced in microsatellites. Proliferating cell nuclear antigen (PCNA), replication protein A (RPA), replication factor C (RFC), and exonuclease I (ExoI).

The high number of repeats in microsatellites make them vulnerable to mutations due to the increased probability of defects during DNA replication and repair. However, microsatellite stability is regulated, and mutations are kept in check through the MMR repair pathway [2,50]. MMR repair consists of three steps: Recognition, excision, and re-synthesis (Figure 1). In brief, MMR corrects DNA mismatches generated during DNA replication, thereby preventing mutations from becoming permanent. Thus, MMR reduces replication-associated defects. This pathway is conserved from bacteria to humans and targets base substitution mismatches and insertion-deletion mismatches (IDLs) [51]. The MMR pathway has been extensively studied and the key players in this pathway are MLH1, MLH3, MSH2, MSH3, MSH6, PMS1, and PMS2 (MutL and MutS homologs) and proliferating cell nuclear antigen (PCNA). The supporting factors include exonuclease I (ExoI), replication protein A (RPA), replication factor C (RFC), DNA polymerase delta, and epsilon as well as DNA ligase I.

The first step in repair is the recognition of the mismatch by MutS homodimer complex. The MutS-alpha is formed by MSH2/MSH6 which recognizes single base mismatches and short insertion-deletion loops, while MutS-beta is formed by MSH2/MSH3 which recognizes IDLs greater than two bases. The MutS recruits PCNA and RFC proteins. RFC loads PCNA, which has an essential role in the excision repair and DNA synthesis process. MutL formed by MLH1 and PMS2 are recruited and they mediate the activation of downstream processes. MutS, MutL, and mismatched DNA form a ternary complex. The ExoI is activated and removes the mismatched base(s). The RPA displaces the mismatch base and also protects the DNA. Subsequently DNA polymerase and ligase complete the resynthesis of the DNA strand [4,52,53].

**Figure 2:**
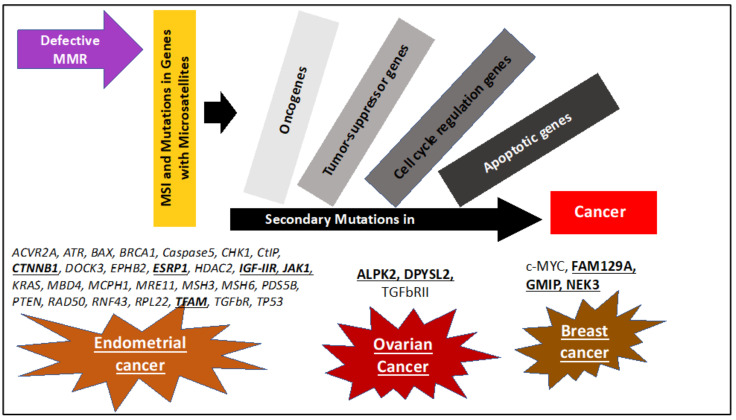
Steps in MMR deficiency (dMMR)/MSI cancer development leading to tumorigenesis. Evidence propose that MSI and the initial mutations cause a cascade of additional mutation in secondary genes in onco-, regulatory, tumor-suppressor, and repair genes. Genes affected are cancer-specific and examples are indicated in the diagram. A cascade etiology would also explain the high mutation rate in dMMR/MSI gynecological cancers. In addition, identifying the genes affected in each specific cancer types will help in understanding better cancer progression and developing markers for effective and timely screening.

Impaired MMR repair due to inherited or spontaneous mutations can give rise to mutations, in particular at microsatellite repeat sequences, causing MSI (Figure 2). MSI is associated with the hypermutator phenotype that is observed in tumors with defective MMR repair system [4]. Defective MMR can be caused by both genetic and epigenetic mechanisms. Individuals with a germline mutation in the MMR genes have an increased risk of carcinogenesis, for example such as patients with Lynch syndrome. The loss of the second allele (loss of heterozygosity (LOH)) due to somatic mutation or epigenetic events further exacerbates the cell dysfunction and leads to tumorigenesis [54]. However, it was reported that in some instances, MMR genes may exhibit haploinsufficiency within a single allele that is sufficient enough to initiate tumorigenesis depending on mutation and affected MMR gene. Epigenetic alterations such as DNA methylation of the *MLH1* is also reported to cause inactivation of MMR system and trigger cancer development [55]. 

It was reported that 77% of sporadic endometrial cancers had MSI due to methylated *MLH1* promoter [10]. In addition, mutations in MMR genes are the second highest cause of hereditary ovarian cancer, accounting for 10–15% of hereditary ovarian cancer cases [5]. This includes mutation in the *MLH1* or *MSH2* gene. A 2006 study reported that Lynch syndrome patients with *MSH6* mutation had a 33% lifetime risk of ovarian cancer [56]. However, these studies are limited by the sample size, population studied, and the methods of investigation, making the exact magnitude of risk still not known [57]. A recent study of Lynch syndrome patients, using multigene panel observed that out of the 528 patients with MMR mutations, 11.9% had breast cancer and 27.3% had colorectal cancer, with *MSH6* and *PMS2* mutations more frequent than *MLH1* and *MSH2* mutations [58]. Though clinical studies evaluating breast cancer risk in patients with Lynch syndrome are not conclusive. It has been suggested that breast cancer risk may vary depending upon the gene affected [59]. It has been observed that mutation in *MSH6* and *PMS2* increases the risk of breast cancer by 30% and 35%, respectively, irrespective of other personal cancer history [59].

## 4. Microsatellites Used as Markers for MSI/dMMR Cancer Diagnosis

MSI is a manifestation of dMMR that results in increased mutation rates throughout the genome, leading to tumorigenesis. Currently, MSI are mainly detected by PCR of microsatellite regions [60]. This MSI typing is used to identify tumors caused by dMMR/MSI by comparing the number of repeats in a panel of microsatellite markers in normal tissue versus tumor tissue from the same individual (Table 1). Alterations in the repeat length of each marker are evidences of MSI. The selection of markers that is used as the gold standard for MSI detection was recommended in 1997 by the National Cancer Institute (NCI) consensus workshop. This marker panel, also known as Bethesda panel, consists of two mononucleotide markers (BAT25 and BAT26) and three dinucleotide markers (D2S123, D5S346 and D17S250) [60,61]. Instability in two or more markers indicates high frequency MSI (MSI-H) tumors while instability in any one marker is suggestive of low frequency MSI (MSI-L) tumors. In addition, MSI markers located in relevant cancer genes have been evaluated for the assessment of MSI status. Since there are evidences for tumor-type specific MSI, separate panels of markers have been now established for some cancer types, for example for prostate cancer [62].

**Table 1:** Details of the markers used for detection of microsatellite instability (MSI) gynecological cancers.

Repeat Type	Marker	Repeat Sequence	Gene	Studied for Detection of MSI	Reference
M O N O N U C L E O T I D E	BAT26	(T)25	*MSH2* gene (*MMR*)	Ovarian cancer, Cervical cancer, Endometrial cancer	[63,64,65,66,67,68,69,70,71,72]
	BAT25	(A)26	*c-kit* gene (oncogene)	Ovarian cancer, Endometrial cancer	[63,65,66,67,68,69,70,71]
	BAT34C4	(T)3C(T)6C(T)17C(T)5C(T)3	*p53*	Endometrial cancer	[68]
	BAT40	(A)40	*3-beta-hydroxysteroid dehydrogenase* gene	Endometrial cancer	[63,68]
	NR-21	(A)21	*SLC7A8*	Ovarian cancer, Endometrial cancer	[63]
	NR-22	(A)22	*Trans-membrane precursor B5*	Ovarian cancer, Endometrial cancer	[63,70]
	NR-24	(T)24	*Zinc finger 2*	Endometrial cancer	[70]
	NR-27	(T)27	*Inhibitor of apoptosis-Protein 1*	Ovarian cancer, Endometrial cancer	[63,70]
	TGFBR-II	(A)10	*TGF-beta receptor*	Ovarian cancer Endometrial cancer	[73,74]
D I N U C L E O T I D E	D2S123	(CA)13(TA)(CA)15	*hMSH2*	Cervical cancer, Endometrial cancer, Ovarian cancer	[65,69,70,71]
	D3S1260	(AGAT)11	*XYLB* gene	Cervical cancer, Endometrial cancer	[63]
	D3S1611	(CA)11	*hMLH1* gene	Breast cancer	[71,75]
	D5S346	(CA)26	*APC*	Cervical cancer, Endometrial cancer, Ovarian cancer	[65,68,69,70,71]
	D10S197	(CA)7…(CA)17	*GAD2* gene	Endometrial cancer, Ovarian cancer	[68,71]
	D11S1318	(CA)15…(CA)5	*eIF3f* gene	Ovarian cancer	[76]
	D11S904	(CA)14(TA)5	*-*	Ovarian cancer	[71,77]
	D17S807	(CA)n	*P53* gene	Breast cancer	[75]
	D17S796	GT)n	*P53* gene	Breast cancer	[75]
	D17S250 (Mfd15)	(TA)7….(CA)24	*BRCA1* gene	Cervical cancer, Endometrial cancer	[65,68,69,70,71]
	D18S55	(GC)5GA(CA)17	*-*	Endometrial cancer	[68]
	NME1		*Nucleoside diphosphate kinase1*	Ovarian cancer	[71]
T R I N U C L E O T I D E	AR	CAG	*Androgen receptor*	Breast cancer	[75]
	DM1	CAG	*Myotonic dystrophy protein kinase*	Ovarian cancer, Breast cancer	[78]
T E T R A N U C L E O T I D E	D2S443	(AAAG)n	-	Ovarian cancer	[72]
	D8S321	(AAAG)12	-	Ovarian cancer	[72]
	D20S82	(AAAG)10	*RM267*	Ovarian cancer	[72]
	DXS981	TATC		Breast cancer, Ovarian cancer	[79]
	DXS6800	(TAGA)x-CA-(GATA)1-GAT-(GATA)y-GG-(TAGA)3-TC-(GATA)3	*X-chromosomal short tandem repeats*	Ovarian cancer	[71]
	MYCL1	(AAAG)21	*MYCL1*	Endometrial cancer	[68]
	UT5037	(AAAG)19	*-*	Ovarian cancer	[72]
	UT5320	(AAAG)21 (AAAG)10	*241A/241B*	Ovarian cancer	[72]
	vWF-a	TCTA	*Von Willebrand factor-alpha*	Ovarian cancer, Breast cancer	[78]
PENTA-NUCLEOTIDE	FMR2	(CCAAA)6(CCAGA)2	*X chromosome*		
	TP53Alu	(AAAAT)8	*p53*	Ovarian cancer	[80]

Whether the markers of the Bethesda panel are sensitive enough to detect MSI in gynecological cancer is still not conclusive. Murphy and Wentzensen compared relative proportion of instability of Bethesda panel markers in colorectal cancer and ovarian cancer [61]. They observed that instability of mononucleotide markers was less frequent than dinucleotide markers in ovarian cancer, which was reverse in comparison to colorectal cancer. Another study concluded that BAT26 is not a suitable marker to detect MSI in cervical cancer patients [81]. Similarly, Ozer et al. reported no MSI in breast cancer patients using the Bethesda microsatellite loci [75]. Depending upon which MSI markers are used, 6–37% of ovarian cancer showed MSI-H phenotype [82]. In these previous reports it is unclear whether the differences in MSI rate in ovarian cancer is due to the choice of loci, or small study sizes. Such findings make it critical to have correct defined panel for each cancer type as it is not clear if the results are biased due to the choice of markers, inadequate number of markers, and/or studied DNA samples. One research group compared concordance between immunohistochemistry and MSI testing (Bethesda panel) for identifying MMR deficiency in epithelial ovarian tumors. They reported poor concordance rates in ovarian cancer (about 68%) as compared to the higher concordance rates (>96%) in colorectal cancer (CRC) [4]. Thus, due to the paucity of studies and data, it is not clear whether the markers in the Bethesda panel are sensitive enough to detect comprehensively MSI in gynecological cancers [61].

Some alternative MSI markers are panels of pentaplex/hexaplex repeats that also contain mononucleotide markers [63,83,84,85]. However, these panels overlook tetranucleotide repeats. Elevated microsatellite alterations at selected tetranucleotides (EMAST) has been reported in various types of cancer including ovarian cancer (0–19%) and endometrial cancer (39%) [79]. Thus, these reports highlight the limitations of these panels for assessing MSI. Wong et al. observed that the NCI recommended panel of markers were not useful for analysis of MSI status in cervical cancer and suggested that using more than five markers can improves the MSI detection [64]. Similarly, it was reported that a MSI pentaplex marker panel was not sensitive and specific in screening gynecological dMMR/MSI cancers [86]. With the advent of next-generation sequencing (NGS), several computational tools for MSI detection were established, however, there is an urgent need to develop MSI panels which are specific and sensitive for each gynecological cancer types.

Recently, framework marker panels have been developed for cervical cancer [87] and ovarian cancer [88]. Some MSI markers used in assessment of gynecological cancer are summarized in Table 1. It seems that markers containing mono- and dinucleotide repeats are more frequently used for gynecological cancers. In addition, target genes with poly(A) and poly(T) repeats are more frequently affected in gynecological cancers (Table 1 and Table 2). Altogether, it is evident from the literature that the sensitivity of MSI detection in cancer is dependent on the choice of the markers, thus, new marker panels for gynecological cancers have to be established. In addition, recent studies have shown that depending upon cancer type MSI tumors are more prone to exhibit mutations in specific genes (Figure 2). Thus, study to identify target genes for MSI will not only help to better understand tumorigenesis but also can be used to develop new markers that can aid in screening cancers for MSI.

## 5. Sequential Steps Leading to MSI/dMMR Cancer Development

To identify disease markers, it is important to understand the disease etiology and the events that lead to MSI/dMMR cancer development. As described before repeat sequences, such as microsatellites, face higher frequency of replication defects that if unrepaired can lead to mutations and alterations in the number of repeats. Postreplication MMR works to maintain genome stability by repairing these errors. A defective MMR system leaves replication defects behind causing MSI. Nevertheless, the link between MSI and cancer development is not well-defined.

Due to MSI, a cascade of events leads to a 100- to 1000-fold increase in the mutation rate, called the hypermutator phenotype [3]. Various genes with microsatellites are observed to have mutations as a result of dMMR/MSI and are believed to be the cancer drivers (Figure 2). For example, microsatellites are present in many regulatory genes, tumor suppressor, pro-apoptotic, and oncogenes, which makes these genes vulnerable to mutations (Table 2). Kawaguchi and colleagues proposed, upon analysis of mutations in 22 patients with sporadic MSI-H endometrial cancer, a novel cascade etiology of carcinogenesis wherein genes affected by MSI could increase in genomic instability and trigger mutagenesis of additional target genes. This causes an accumulation of mutations and deficiencies of other cancer-related genes [73]. Identifying these genes would be very helpful for detection and treatment of gynecological dMMR/MSI cancers.

Mutational characteristic and target genes differ between various dMMR/MSI cancer types. Affected genes previously reported in colon and gastric cancers have been observed to have a low mutation rate in gynecological cancers. For example, it was shown that frameshift MSI in *BRAF, TGFbeta RII,* and *BCL-10* genes are common in colorectal and gastric cancers, but occur infrequently in endometrial or ovarian cancers [54,74,89,90,91]. On the other hand, 40% of endometrial cancers patients show mutations in *JAKI* gene as compared colorectal cancer patients (less than 10%) [92]. Furthermore, recently Wang et al. observed that the *RPL22* gene was frequently mutated in MSI endometroid cases (50%) in contrast to *TP53* gene, which was mutated in 40% of microsatellite stable (MSS) endometroid tumors [93]. Hence there is a need to identify specific genes that are affected in each cancer types. This will aid in understanding cancer progression as well as in developing markers for effective cancer screening.

In cases of endometrial cancer, the *ACVR2A* gene is shown to have strong predictive specificity for MSI-H tumors. The same study found also that *JAK1*, *TFAM,* and *SMC6* genes are affected in endometrial cancer cells [54]. In addition, genes reported to be frequently mutated in MSI endometroid cases include *RPL22*, *PTEN*, *KRAS*, *ATR*, *CHK1*, *CDC5*, *Caspase5,* and *BAX* gene [92,93,94,95,96]. *JAK1* mutations are also observed in cervical cancer cases caused by MSI [94]. Furthermore, the authors uncovered new genes affected by frameshift MSI events, including *FAM129A*, *GMIP,* and *NEK3* genes in breast cancer and *DPYSL2* and *ALPK2* genes in ovarian cancer [54]. These genes can be potentially used as markers for MSI detection (Table 2).

In summary, carcinogenesis in dMMR/MSI tumors can be explained as a cascade wherein mutations in MMR and subsequent MSI leads to mutagenesis of other regulatory genes, oncogenes, tumor-suppressor genes, and pro-apoptotic genes that can trigger oncogenesis (Figure 2). It is important to highlight that many but not all target genes affected by MSI harbor microsatellites. In Table 2 are listed target genes that are affected in gynecological MSI cancers. Between studies differences in percentage of prevalence of some target genes were reported. This could be attributed to lower sample size and other characteristics such as ethnicity of the patients. Further analysis of dMMR/MSI gynecological cancers and identification of new target genes will give a better idea about the cancer development and aid in better screening of dMMR/MSI gynecological cancers.

**Table 2:** Target genes that harbor MSI in gynecological cancer.

Functional Group	Gene	Role	Repeat Sequence if Present	% Frequency of Mutation in MSI-H			
				Endometrial Cancers	Ovarian Cancers	Breast Cancer	Non-Gynecological Cancers
Cell regulation/signaling	*ACVR2A*	Member of TGF-beta signaling pathway. Role in cell growth and tumor metastasis	2(A)8	19% [97]			CRC 80% Stomach 75% [97]
	*CHK1*	DNA damage response	(A)9	29% [98]			
	*c-MYC*	Cell division	(GT)n–(GC)n			20% [44]	
	*DPYSL2*	Microtubule function. May play role in endocytosis	(CT)11		59% [54]		
	*ESRP1*	Protein-splicing regulator. May contribute to mesenchymal transition	(GGT)n	20% [97]			
	*GMIP*	Cell growth and survival. *Ras* pathway				10% [54]	
	*HDAC2*	Histone deacetylase	(A)n	11% [73]			
	*IGFRIIR*		(G)8	14% [98]			
	*MBD4*	Methyl CpG	(A)10)	31.8% [73]			
	*NEK3*	Mitotic regulator	(A)8			6% [54]	
	*PDS5B*	DNA damage repair	(A)9	15% [69]			CRC 28% [69]
	*PTEN*	DNA damage response	(A)6	15.8% [73], 88% [54]			CRC 28% [99]
	*RNF43*	Involved in controlling cell proliferation Negative regulator of WNT pathway.	(G)7	23% [97]			CRC 40% Stomach 35% [97]
	*RPL22*	Protein synthesis	(A)8	37% [54] 50% [93] 52% [100]			CRC 80% [100]
	*TGFBR*	TGF-beta receptor	(A)10	36.3% [73] 5% [54]			CRC 90% [73]
Oncogenes	*ARID1A*	Tumor suppressor gene. Regulates transcription of certain genes by altering the chromatin structure around those genes	(AT)n	37% [101]			
	*JAK1*	Oncogene. Modulates IFN-gamma signaling pathway and enables tumor immune evasion Promotes tumor survival	(T)7, (T)8, (G)7	21% [97] 35% [102]			
	*KRAS*	Oncogene		35% [54]			CRC 31% [99]
	*TP53*	Tumor suppressor	TP53 ALU (A)n (AAAAT)8	40% [93]	21%		CRC 31% [99]
WNT pathway	*CTNNB1*	Member of WNT pathway	(A)n	30% [97]			CRC 6% [69]
	*DOCK3*	Protein dedicator of cytokinesis 3 Inhibits WNT pathway		23% [97]			Stomach 40% [97]
	*EPHB2*	Member of WNT pathway	(A)9	9% [73] 14% [103]			Gastric 39% [103]
Apoptosis pathway	*ALPK2*	Apoptosis and DNA Repair	(T)3		17% [54]		
	*BAX*	Pro-apoptotic factor	(G)8	22.7% [73], 16% [96] 43% [98]			CRC 45% [73]
	*Caspase 5*	Pro-apoptotic factor	(A)10	4.5% [73], 5% [96] EC- 28% [104]			Stomach 44% CRC 62% [104]
	*FAM129A*	Apoptosis regulator, Anti-apoptotic	-			12% [54]	
	*TFAM*	Apoptosis regulator, DNA damage repair	(A)10	20% [69]			
MMR genes	*hMSH6*	Repair genes	(C)8	30% [73]			
	*hMSH3*	Repair genes	(A)8	9% [73]			
DNA repair	*ATR*	DNA damage checkpoint	(A)10	15% [49]			
	*BRCA1*	Tumor suppressor gene, DNA repair	(TA)7 (CA)24 Flanking sequences	15% [49]			
	*CtIP*	Promotes the resection of DNA double-strand breaks	(T)9	12% [49]			
	*MCPH1*	DNA damage response protein	(A)9	12% [49]			CRC 9.7% [105]
	*MRE11*	Double Strand Break Repair Nuclease	(T)11	15% [49] 50% [106]			CRC 83% [106]
	*RAD50*	Double Strand Break Repair Protein	(A)9	17% [49]			CRC 46% [107]
Other	*PIK13CA*	Role in protein kinase B signaling	-	54% [101]			
	*PIK3RI*	Role in the metabolic actions of insulin	-	40% [101]			

CRC: Colorectal cancer.

## 6. Sporadic Malignancies Caused by MSI/dMMR

In tissues with a high cell proliferation, there are more opportunities for DNA mutations to be inserted due to frequent DNA replication. Thus, it is the tissues with the highest cell turnover which are the most susceptible to MMR proteins deficiency. Cell turnover in the gastrointestinal mucosa and endometrial tissue are among the highest in the body, which would be one explanation for why these two organs are particularly susceptible to develop MSI that results in malignancy [1,108]. Deficient MMR function and MSI is observed in 20% to 30% of patients with endometrial cancer [73,93,109,110,111]. In about 90% of those cases, carcinogenesis was due to a sporadic gene mutation [112,113].

The primary risk factor for type 1 endometrial cancer that are estrogen dependent is an increased level of estrogen [114]. Women with this exposure who are at increased risk for endometrial cancer include women with early menarche, late menopause, obesity, chronic anovulation, tamoxifen use, and estrogen-secreting tumors. However, the relationship between estrogen exposure and endometrial cancers with MSI is less clear [115,116]. Estrogen binding to the estrogen receptor-β has been shown to upregulate MMR protein activity through enhanced MLH1 and MLH2 expression in vitro and in vivo [116,117]. However, the risk factors identified for the development of endometrial cancer persist whether MMR proteins are deficient or not [118]. One explanation could be that estrogen encourages the growth of endometrial cells in the uterus, thus causing higher cell proliferation and higher risk for replication errors.

The Society of Gynecologic Oncology recommends screening all endometrial cancers for MMR deficiency [119]. Other gynecologic malignancies in which MSI has been identified include uterine carcinosarcoma, cervical carcinoma, and ovarian carcinoma. In these cancers, the overall uncommon occurrence of MSI means that testing for MSI is not routinely performed and does not guide therapeutic management at this time [1]. Tumor testing for defective MMR is performed using MSI typing or immunohistochemistry. Immunohistochemistry is performed using antibodies that bind to MMR proteins (MLH1, MSH2, MSH6, and PMS2) to stain for the expression level of these MMR proteins [120]. A mutation which results in a deficient or absent MMR protein will appear as a lack of staining for that particular protein. When deficient MLH1 is identified, testing for *MLH1* promoter hypermethylation is performed to determine the quantity of DNA which is methylated in the promoter region of the *MLH1* gene. MSI testing can be performed alone or in combination with immunostaining to evaluate for genetic mutations [113,121]. A positive result for immunohistochemistry, with negative hypermethylation testing, or a positive result for MSI will then trigger evaluation for germline mutations in MMR genes to rule out Lynch syndrome.

Among all patients who present with endometrial cancer, the presence of MSI due to a somatic mutation does not appear to affect overall survival with the use of conventional treatment modalities. Yet, there is evidence that survival is improved in early stage tumors with high levels of MSI that are treated with adjuvant radiotherapy [122,123]. However, emerging immunotherapies are being developed which may provide additional pharmacologic treatment options for patients with MSI malignancies.

## 7. Inherited Malignancies Caused by MSI/dMMR-Lynch Syndrome

There is a great importance to identify MMR deficient tumors due to germline mutations, rather than somatic mutations alone. Patients with a germline mutation of a MMR gene have an increased lifetime risk of several malignancies due to MSI. Additionally, the identification of patients with Lynch syndrome allows for genetic testing and cancer prevention strategies in the patient’s family members who may have inherited the same mutation. The overall risk varies significantly depending on which gene is mutated. There is a cumulative risk for any cancer at age 70 as low as 18% for patients with *PMS2* mutations and as high as 72% for both patients with a *MLH1* mutation or *MSH2* mutation [124]. Endometrial and colon cancers are among the most common cancers in patients with Lynch syndrome [125]. The incidence for endometrial cancer by age 40 is 2 to 3%. However, by age 70, the cumulative risk increases significantly, but varies depending on the affected gene (Table 3) [124,125,126]. The incidence of cervical carcinoma and uterine sarcoma is not well described in Lynch syndrome and therefore it is not clear if the risk is increased compared to the general population.

Patients with Lynch syndrome are usually identified after genetic screening and MSI-H diagnosis, or because of a family history of Lynch syndrome-associated cancers. Once Lynch syndrome has been identified, patients are recommended to receive counseling about their increased risk for multiple cancers including endometrial, colorectal, ovarian, gastric, hepatobiliary, urinary tract, brain, and skin cancers. They should additionally be made aware of the recommended screening guidelines for cancers in which an effective method exists. For gynecologic cancers, the American College of Obstetricians and Gynecologists (ACOG) recommend screening for endometrial cancer with an endometrial biopsy every 1 to 2 years, beginning at age 30 to 35 years, indefinitely or until a risk-reducing hysterectomy is performed. They should also keep a menstrual calendar and report any abnormal bleeding to their physician [127]. To date, no effective screening method to detect ovarian cancer has been demonstrated for patients with Lynch syndrome and therefore it is not currently recommended by ACOG [128]. Thus, as mentioned before underlining the importance for identification of MSI markers for gynecological dMMR/MSI cancers.

In addition, the option of risk-reducing surgery by prophylactic total hysterectomy, bilateral salpingectomy with or without bilateral oophorectomy should be discussed. This effective strategy was shown to decrease the risk of endometrial cancer to 0% compared to 33% in Lynch syndrome control patients after a 7 years follow-up [129]. This option should be considered once childbearing is complete or by age 40 given the increased endometrial and ovarian cancer incidence that occurs in these patients between ages 40 to 70. However, although risk-reducing surgery is the most effective way to avoid cancer development, this is an invasive procedure which holds the risk of complications and could lead to adverse outcomes. A bilateral oophorectomy, particularly in pre- or peri-menopausal women can result in additional health problems like a higher risk of cardiovascular disease and a decrease in bone mineral density [130,131].

## 8. Immunotherapy for MSI/dMMR Gynecological Cancers

**Figure 3:**
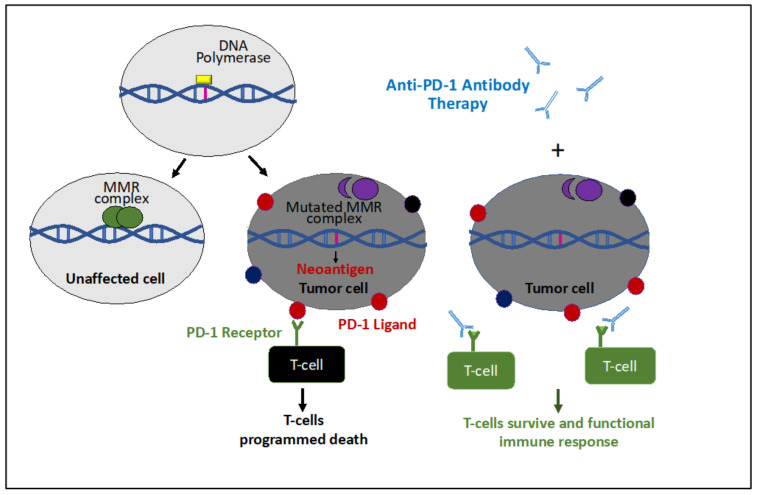
Illustration of effect of the anti-programmed cell death-1 (anti-PD-1) antibody used for treatment of MSI/MMR gynecological cancer: In human cells the DNA polymerase can slip and insert or delete nucleotides at the repetitive DNA sequences, such as microsatellites. If these replication errors are not repaired by the repair machinery due to a defective MMR, it can lead to MSI. Translation of such genes with MSI can result in creation of novel peptide sequences, such as neoantigens (e.g., PD-ligands). Thus, these ligands on the tumor cell can trigger cell death of T cells and so evade an immune response. The anti-PD-1 antibodies bind programmed cell death-1 (PD-1) receptor and can prevent activation of programmed cell death by the PD-1 ligand.

Cancer immunotherapy is a new rapidly advancing field of cancer therapy, joining surgery, cytotoxic chemotherapy, radiation, and targeted therapy. The concept behind immunotherapy is to take advantage of the immune response to tumor cells in order to better target the malignant tissue. One component of the normal immune response to malignancy is T-cell activation against tumor cells. This mechanism has several checkpoints that are put in place by the immune system in an attempt to keep this response balanced and prevent over-activation and self-induced harm. One such a checkpoint is programmed immune cell death. There is a receptor on the surface of T-cells, the programmed cell death-1 (PD-1) receptor, which becomes activated by PD-1 ligand on the surface of tumor cells (Figure 3) or by adjacent immune cells. Binding of this ligand to the PD-1 receptor signals the cell to undergo apoptosis. Anti-PD-1 immunotherapy is a pharmacologic antibody which has been developed to target this checkpoint and promote continued T-cell activity to prevent apoptosis of these cells.

Anti-PD-1 immunotherapy has been shown to be effective across a wide range of cancers. Biomarkers have been identified for better prediction which cancers might respond to anti-programmed cell death-1 (anti-PD-1) immunotherapy [132]. These biomarkers include analysis of the expression of PD-1 receptors and their ligands, high tumor mutational burden, and the presence of MSI [133,134,135,136]. The sequence of events which leads to the presence of these biomarkers in MMR deficiency cancers is caused by the uncorrected mutations that occur when MMR proteins are deficient, which can lead a high tumor mutational burden. DNA mutations can then lead to the expression of novel proteins (neoantigens), which can cause an immune response and upregulation of PD-1 ligand [137] (Figure 3). In the anti-PD-1 immunotherapy the antibody prevents binding of the PD-1 receptor on the surface of T-cells to the tumor cells and thus apoptosis of the T-cells. This therapy is unique, in that it is not specific for a tissue-type, but instead is specific for a biomarker which can be present in almost all tissue types.

While MMR deficiency can lead to malignancies in many different types of tissue, among gynecologic malignancies that are known to occur due to this mutation, endometrial cancer is the most common and the most likely to be tested for MSI [1,108]. In addition, the presence of tumor-infiltrating lymphocytes (CD8+) and PD-L1 expression are observed to be significantly higher in the MSI group compared to the microsatellite-stable group. These results suggest that immune checkpoint inhibitors (anti-PD-L1 antibody) could be effective in endometrial cancers with MSI. The presence of MSI may be a biomarker for good response to PD-L1 immunotherapy in endometrial cancer [138]. Initial studies in MMR deficient gynecologic cancers have shown that these tumors do respond to anti-PD-1 immunotherapy, however these initial studies were designed to include any cancer type with known MMR deficiency, and therefore the overall numbers of gynecologic cancers included were low [139,140].

In the largest of these two studies, patients with non-colorectal MMR deficient cancers which previously failed conventional treatment and had evidence of disease progression were enrolled to receive an anti-PD-1 immunotherapy. The cohort included 27 tumor types and included 49 cases of endometrial cancer, 15 cases of ovarian cancer, 6 cases of cervical cancer, 1 vaginal cancer case, and 1 vulvar cancer case. Among the entire cohort, an objective response was observed in 36.3% of patients with a median overall survival of 23.5 months. The tumor types with the highest enrollment were also individually analyzed. Among endometrial cancers, the median progression free survival was 25.7 months and among ovarian cancers a median progression free survival of 2.3 months was observed (Table 4). Importantly, while the number of patients with a complete response to treatment was low, even when a partial response was observed, it was often prolonged and durable. These results suggest that immune checkpoint inhibitors are useful as an adjunctive treatment for patients with MMR deficient gynecologic tumors, yet their exact role in treatment must be further explored.

Additional prospective data assessing the effect of anti-PD-1 immunotherapy in gynecologic malignancies with dMMR is needed in order to confirm which gynecologic cancer types respond to this treatment and at which point in treatment this immunotherapy is most effective. There is currently one anti-PD-1 monoclonal antibody, pembrolizumab, that is approved by the US Food and Drug Administration (www.fda.gov). This immunotherapy is approved for use with all solid tumors that are MMR deficient or have MSI-H which have progressed following prior treatment and no satisfactory alternate treatment options are available.

## 9. Conclusions

MMR deficiency leading to MSI and subsequent malignancy has been identified in various cancer types, however it is most prevalent among gynecologic cancers, particularly endometrial cancer and colorectal cancer. DNA replication of microsatellites is prone to hindrance due to the inherent nature of the repeats and their ability to form secondary DNA structures. This makes genes containing microsatellites susceptible for genomic instability and has been proposed as an early step in carcinogenesis. Defective MMR can lead to mismatch-induced frameshift mutations in genes containing microsatellites and influence their expression level in the cell. In patients with Lynch syndrome inherited genetic and/or epigenetic mechanisms are responsible for the loss of MMR gene expression and MSI. Microsatellites are present in many regulatory, pro-apoptotic, and tumor suppressor genes. Thus, MSI can lead to genomic instability and mutations for example in genes important for DNA damage repair and regulation of cell growth.

Various MSI markers are routinely used for testing to identify MSI-H cancers, to help to treat patients. However, literature indicates that most of the MSI testing is limited to certain types of cancer such as endometrial and colorectal cancer. In endometrial cancer, the identification of dMMR/MSI tumors is helpful to identify patients with Lynch syndrome who will benefit from cancer screening strategies for other cancers associated with this syndrome. However, from a treatment perspective, dMMR/MSI gynecologic cancers are managed with the same first line treatment protocol like gynecologic cancers without dMMR/MSI. The recent development of an immunotherapy, which targets PD-1 receptors to prolong the immune response against the tumor cells, adds a new tool to use against dMMR/MSI cancers. Anti-PD-1 antibody immunotherapy is currently approved for use in dMMR/MSI cancers which have failed traditional treatment strategies. It is not currently known whether immunotherapy would add survival benefit when used earlier in the treatment of gynecologic dMMR/MSI cancers. Studies are needed to better understand the optimal time point to use immunotherapy for treatment of dMMR/MSI gynecologic cancers.

Compared with colorectal cancer, survival and treatment response in MMR defective gynecological cancer are hugely under-investigated. There is a need to develop MSI marker panels which are specific to gynecological cancers for effective screening and treatment. In addition, investigating the role of dMMR/MSI will not only provide insight into the pathogenesis of gynecological cancers, it could also influence treatment and survival. Elucidation of the pathways leading to dMMR/MSI gynecological cancers will help develop better predictive models of cancer progression and novel therapeutic approaches.

## Figures and Tables

**Table 3:** Cumulative gynecologic cancer risk at age 70 by MMR gene germline mutation type.

Gene Mutation	Endometrial Cancer	Ovarian Cancer
*MLH1*	34–54%	11%
MSH2 *	21–51%	15%
*MSH6*	16–49%	0–1%
*PMS2*	13–24%	0–1%

* +/− *EPCAM* mutation.

**Table 4:** Response to pembrolizumab among gynecologic cancer subtypes from the phase II KEYNOTE-158 study.

Cancer Type	Number Enrolled (*n*)	Complete Response (*n*) (%)	Partial Response (*n*) (%)	Objective Response Rate, Months (95% CI)	Median Progression Free Survival, Months (95% CI)
Endometrial	49	8 (16.3%)	20 (40.8%)	57.1 (42.2–71.2)	25.7 (4.9–DNR)
Ovarian	15	3 (20%)	2 (13.3%)	33.3 (11.8–61.6)	2.3 (1.9–6.2)
Cervical	6	NR	NR	NR	NR
Vaginal	1	NR	NR	NR	NR
Vulvar	1	NR	NR	NR	NR

KEYNOTE-158 [140] was a nonrandomized, open-label, multisite phase II study that enrolled patients with advanced high frequency MSI (MSI-H)/dMMR non-colorectal cancer. DNR: Did not reach; NR: Not reported. Complete and Partial Response: Per RECIST version 1.1 and determined by an independent radiologist.
